# Do corticosteroids affect immunotherapy efficacy in malignancy? – A systematic review

**DOI:** 10.1002/cam4.70254

**Published:** 2024-09-24

**Authors:** Yoni Byron, Sonya Yegorova‐Lee, Martin Tio

**Affiliations:** ^1^ Alan Walker Cancer Centre Royal Darwin Hospital Darwin 0810 Northern Territory Australia; ^2^ School of Medicine Griffith University Gold Coast Queensland Australia; ^3^ Department of Medical Oncology Tweed Hospital Tweed Heads New South Wales Australia

**Keywords:** cancer management, clinical management, corticosteroids, immunotherapy, meta‐analysis, prognostic factor

## Abstract

**Background:**

Early studies indicated that corticosteroids may limit the survival benefit from immunotherapy. We conducted this systematic review to evaluate the effect corticosteroids have on immunotherapy in patients with malignancy, when adjusted for potentially confounding effects of corticosteroids given for palliative indications.

**Methods:**

Three electronic databases (PubMed, Embase and Medline) were searched on 1 February 2023. Studies that measured response or survival to immunotherapy in people receiving corticosteroids for non‐cancer indications compared to either no corticosteroids or corticosteroids for cancer‐related indications were included. Studies exclusively evaluating the effect of corticosteroids administered for immune‐related adverse events (irAE) were excluded to avoid immortal time bias. Pooled odds and hazard ratios with 95% confidence intervals (CI) were calculated using a random effects model. Study heterogeneity was assessed using the *I* ^2^ statistic, and publication bias was evaluated by funnel plot and Egger's regression model.

**Results:**

Eight thousand four hundred and twenty‐six titles were identified on our search. Eight studies met our inclusion criteria for meta‐analysis. Administration of corticosteroids does not have a statistically significant effect on survival and response to immunotherapy when administered for non‐cancer‐related indications, with a pooled odds ratio for overall response rate 1.01 (95% CI 0.64–1.60); pooled hazard ratio (HR) for progression free survival 0.87 (95% CI 0.68–1.12); and pooled HR for overall survival 0.79 (95% CI 0.59–1.05).

**Conclusion:**

This systematic review indicates that administration of corticosteroids does not affect response to immunotherapy nor survival outcomes, when removing confounding palliative corticosteroid indications. These results are limited by the retrospective nature of the studies included, small sample sizes, lack of information about corticosteroid dosing and the inclusion of irAE in two of the studies which could bias the results.

## Graphical abstract

Systemic review and meta‐analysis of retrospective trials assessing the effect of corticosteroids on response rates in patients with malignancy being treated with immunotherapy. When indications for corticosteroids are not associated with a poor prognosis, prescribing corticosteroids does not seem to affect response rates to immunotherapy or overall survival.

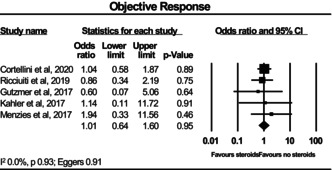

## 1 INTRODUCTION

Immune checkpoint inhibitors (ICI) have revolutionised medical care for patients with malignant diseases, with prolonged responses observed more frequently than with chemotherapy alone. Despite impressive results seen with ICI, most patients do not achieve a sustained clinical response and markers that could predict response, or overcome resistance mechanisms are highly valuable. Corticosteroids are predominantly used for immunosuppression or modulating toxic effect from ICI, thus raising a concern that corticosteroids might also diminish the anti‐cancer effect.

Several studies reported impaired efficacy for ICI in patients also treated with corticosteroids.^1^, ^2^, ^3^, ^4^, ^5^, ^6^, ^7^, ^8^ Kartolo et al. reported a dose‐dependent effect with decreased survival seen at doses of corticosteroids only above 50 mg per day of prednisolone equivalent,^5^ but other reports described a negative effect with prednisolone equivalent dose of 10 mg per day.^1^, ^2^, ^6^, ^9^, ^10^, ^11^, ^12^, ^13^ These reports led clinicians to aggressively down titrate corticosteroid doses prior to ICI, or delay initiation of ICI. Iglesias–Santamaría et al. however, reported in 2020 no effect on immunotherapy by concurrent corticosteroids, but noted a negative impact with concurrent use of opioids.^14^

Use of corticosteroids in patients with cancer is often associated with poor performance status,^15^, ^16^, ^17^, ^18^ and a report by Facchinetti et al. suggested that people with poor performance status have poorer prognosis with immunotherapy,^19^ which could suggest that the observed interaction of corticosteroids with ICI might be confounded.

Several inconsistencies regarding the potential interaction between ICI and corticosteroids need discussion. Firstly, corticosteroids as premedication in landmark trials when ICI was combined with chemotherapy, did not report negative impact on survival in those populations.^20^, ^21^, ^22^ Secondly, there are conflicting results about the association between the effects of corticosteroids on survival when used for the treatment of irAE. Mouri et al. reported a negative effect from use of corticosteroids to treat irAE on both overall survival (OS) and progression free survival (PFS),^23^ whereas Bruyère et al. reported a negative effect on PFS, but not on OS^24^ and multiple other studies reported that systemic corticosteroids for treatment of irAE do not diminish the effect of ICI.^25^, ^26^, ^27^, ^28^, ^29^, ^30^, ^31^, ^32^ Thirdly, several studies showed that ICI are effective in autoimmune conditions, even when requiring systemic corticosteroids.^33^, ^34^, ^35^ Lastly, Ricciuti et al. reported that negative impact on survival was observed in patients prescribed corticosteroids particularly for palliative or cancer related indications.^36^ Several other studies were found that reported a lack of statistically significant impact on survival for patients treated with corticosteroids and ICI, when the steroids were prescribed for non‐cancer related indications.^34^, ^36^, ^37^, ^38^, ^39^, ^40^, ^41^, ^42^

In this systematic review we propose that the negative impact of corticosteroids on ICI therapy is not causative, but rather a confounding effect due to the correlation of corticosteroid therapy with poor performance status or an aggressive disease phenotype. We conducted a systematic literature review for articles that reported comparison of parameters of efficacy such as OS, PFS or response rate in patients treated with ICI in combination with corticosteroids, and detailed the indication for which the corticosteroids were prescribed. Studies looking at such survival parameters with regard to systemic corticosteroid therapy exclusively for irAE were excluded due to the possible effect of immortal time bias (ITB) on survival.^43^, ^44^ While other systematic reviews were done on this topic, most were of a qualitative nature.^45^, ^46^, ^47^ Wang et al. published a similar analysis in 2021^8^ but did not include smaller studies that only reported response rates, and since their report another study was published^42^ and is therefore worth updating for a stronger result.

## 2 METHODS

### 2.1 Eligibility criteria

Any phase II/III randomised control trials, case control studies and cohort studies were considered for inclusion. Only full text articles were eligible, abstracts and conference reports were excluded. Studies had to describe any solid or haematological malignancy in human patients with any type of ICI, with information regarding indications for steroid administration stratified by cancer‐related and non‐cancer‐related. The main clinical outcomes in the studies had to be reported as OS, PFS or objective response rate (ORR). Studies that did not report any of these outcomes but had available information regarding the response of each participant in the cohort were also included and response rate was calculated. Studies were ineligible for inclusion if they were case series or case reports, used immunotherapy for non‐cancer indications or did not report the indications for corticosteroid use. Animal non‐human studies were also excluded. Studies that exclusively evaluated the effects of corticosteroids on survival when prescribed for management of irAE were excluded as it was previously reported to not affect the survival outcome of patients, and including data from such studies would likely cause an ITB in favour of the hypothesis.

### 2.2 Search strategy

Our systematic review was performed following the preferred reporting items for systematic reviews and meta‐analyses (PRISMA) statement.^48^ Two reviewers (YB and SYL) searched MEDLINE, EMBASE and PubMed databases from inception to 1 February 2023. The search terms [(pembrolizumab OR ipilimumab OR nivolumab OR durvalumab OR avelumab OR atezolizumab OR cemiplimab OR tremelimumab OR dostarlimab OR spartalizumab OR camrelizumab OR sintilimab OR tislelizumab OR toripalimab OR ctla4 OR PD1 OR PD‐1 OR PD‐L1 OR ‘immune checkpoint inhibitors’) AND (glucocorticoids OR steroids OR glucocorticosteroids OR prednisolone OR prednisone OR dexamethasone OR methylprednisolone OR hydrocortisone OR cortisone OR beclomethasone OR betamethasone OR triamcinolone) AND (malignancy OR cancer OR neoplasms)] were used. No language limitations were placed. All included studies were searched via Google Scholar for citing references, and the reference lists of included studies were hand searched. Disagreements were resolved by a third reviewer (MT).

### 2.3 Study selection

Eligible studies were included if the following were described: (1) An interventional or observational study design of human patients with any malignancy treated with immune‐checkpoint inhibitors; (2) Reported risk ratios and 95% confidence intervals (CI) of OS, PFS or ORR comparing patients requiring corticosteroids for cancer‐unrelated reasons versus corticosteroids for cancer related reasons, or versus no corticosteroids. Studies that did not report risk ratios and 95% CI but reported data such that risk ratios and 95% CI could be manually calculated, were also included.

Studies that exclusively reported risk ratios of patient outcomes with corticosteroids for immune‐related adverse events were excluded, due to the potential for ITB.^43^, ^44^

### 2.4 Data extraction

Two reviewers (YB and SYL) extracted data from included studies. First author name, year of publication, study design, sample size, malignancy type, stage of malignancy, immune‐checkpoint inhibitor type, steroid indication and comparison group and reported outcomes were extracted.

### 2.5 Statistical analysis

Pooled hazard ratios and 95% CI for OS and PFS were calculated using a random‐effects model. A pooled odds ratio and 95% CI were calculated using a random‐effects model for objective response rate.^49^ Publication bias was examined using Egger's regression test.^50^ Study heterogeneity was measured using the *I* ^2^ statistic.^51^ A *p* < 0.05 was defined as statistically significant. Statistical analyses were performed using Comprehensive Meta‐analysis version 3.3.070, Englewood, NJ, USA (2014).

## 3 RESULTS

**FIGURE 1:**
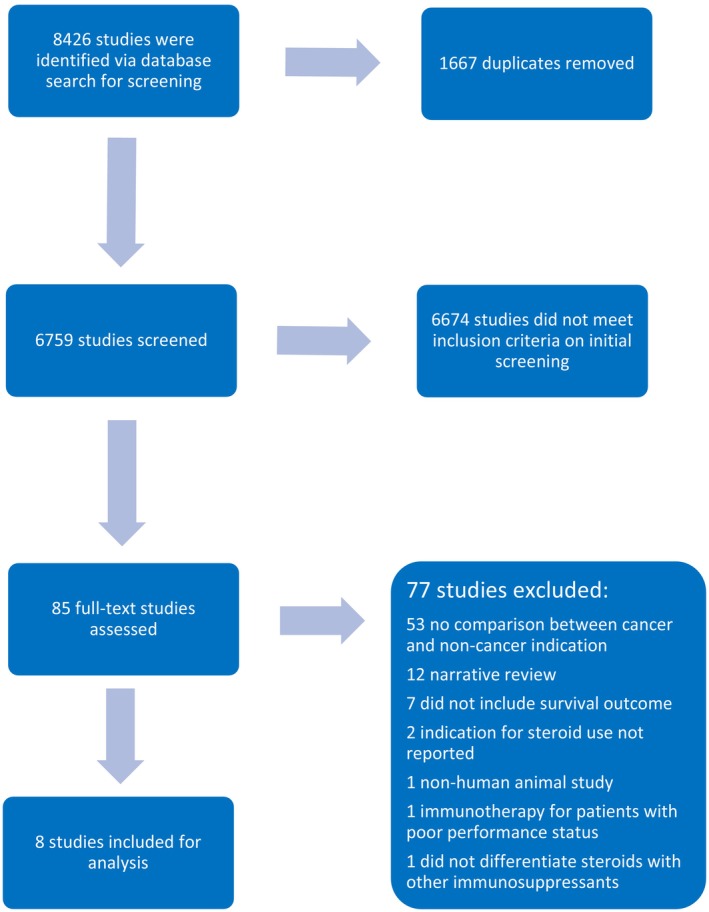
Flow diagram for studies selected.

Eight thousand four hundred and twenty‐six citations were identified in our search, for which eight studies were included for meta‐analysis (Figure 1).^34^, ^36^, ^37^, ^38^, ^39^, ^40^, ^41^, ^42^ Table 1 shows the characteristics of the included studies. All studies were retrospective observational studies, with five reporting objective response rates,^34^, ^36^, ^37^, ^39^, ^40^ and five reporting both OS and PFS.^36^, ^37^, ^38^, ^41^, ^42^ The majority of studies analysed patients with stage IV malignancies. Four studies compared patients without systemic corticosteroids versus those with systemic corticosteroids, while four studies analysed patients with <10 mg equivalent of prednisolone versus patients with ≥10 mg equivalent of prednisolone.^36^, ^37^, ^41^, ^42^ Only two of the selected studies assessed response differences due to baseline or concurrent corticosteroid use,^38^, ^41^ due to the small number of patients in those studies, this factor was not assessed on this analysis.

**TABLE 1:** Characteristics of included studies.

Study	Study type	Single/multi centre	Total no of pts	Cancer	Stage	Immunotherapy	Steroid indication and doses of groups analysed Group 1: no/minimal steroids Group 2: steroids given with ICI	Outcome reported (Group 1 vs.Group 2)
Gutzmer (2017)	Retrospective observational study	Multi‐centre	19	Melanoma	Metastatic	Pembrolizumab Nivolumab	No systemic steroids at start of treatment (*n* = 14) Steroid indications: autoimmune disease (*n* = 5) Information about doses not provided	ORR 29% versus 40%
Kähler (2017)	Retrospective observational study	Multi‐centre	41	Melanoma	Metastatic	Ipilimumab	No systemic steroids at start of treatment (*n* = 32) Steroid indications: autoimmune disease (*n* = 9) Information about doses not provided	ORR 19% versus 33%
Menzies (2017)	Retrospective observational study	Multi‐centre	39	Melanoma	Information not available	Pembrolizumab Nivolumab Prior ipilimumab 80%	No immunosuppression (*n* = 32) Steroid indications: autoimmune disease (*n* = 7) Information about doses not provided	ORR 44% versus 28%
Ricciuti (2019)	Retrospective observational study	Single centre (Dana‐Farber Institute)	650	NSCLC	IV	PD‐1 or PD‐L1 inhibition as monotherapy (94.6%) or in combination with CTLA‐4 (5.4%)	<10 mg prednisolone equivalent per day (*n* = 557) ≥10 mg prednisolone equivalent per day (*n* = 27) Indications: pneumonitis (*n* = 7), ECOPD (*n* = 6), RhA (*n* = 4), contrast prophylaxis (*n* = 4), dermatitis (*n* = 1), renal transplant (*n* = 1), Sjögren (*n* = 1) and sciatica (*n* = 1)	OS: HR 0.91 (95% CI 0.47–1.78) PFS: HR 0.62 (95% CI 0.33–1.17) ORR: 19.7% versus 22.2%
Pinatio (2020)	Retrospective observational study	Multi‐centre	304	HCC	BCLC stage A‐B (24%) C (76%)	279 (92%): single‐agent anti PD‐(L)1 25 (8%): combined PD‐1/CTLA‐4 ICI	<10 mg prednisolone equivalent per day (*n* = 226) ≥10 mg prednisolone equivalent per day (*n* = 70) Indications: procedure/prophylaxis (*n* = 37), comorbidity (*n* = 6) and irAE (*n* = 27)	OS HR 0.40 (95% CI 0.19–0.85) PFS HR 0.60 (95% CI 0.33–1.10)
De Giglio (2020)	Retrospective observational study	Multi‐centre	299	NSCLC	IIIB (0.7%) IV (99.3%)	Immune checkpoint inhibitors	No steroids (*n* = 250) Median dose 50 mg prednisolone equivalent (*n* = 11) Steroid indications: irAE (54.6%), pneumonia + ECOPD (27.1%)	OS HR 0.80 (95% CI 0.35–1.84) PFS HR 0.98 (95% CI 0.49–1.94)
Cortellini (2020)	Retrospective observational study	Multi‐centre	960	NSCLC 52.2% Melanoma 26% RCC 18.3% Other 3.6%	IV	Pembrolizumab 33.9% Nivolumab 60.6% Atezolizumab 3.2% Other 2.3%	<10 mg prednisolone equivalent per day (*n* = 715) ≥10 mg prednisolone equivalent per day (*n* = 50) Steroid indications: inflammatory process unrelated to cancer	OS HR 0.85 (95% CI 0.55–1.32) PFS HR 0.96 (95% CI 0.68–1.36) ORR 41% versus 40%
Skribek (2021)	Retrospective observational study	Single centre	196	NSCLC	IV	Anti PD‐1 or anti‐PD‐L1 agents	<10 mg prednisolone equivalent per day for <10 days (*n* = 104) ≥10 mg prednisolone equivalent per day (*n* = 27) Steroid indications: non‐cancer‐related indications (exact indications not detailed)	OS HR 1.03 (95% CI 0.48–2.23) PFS HR 1.24 (95% CI 0.68–2.26)

Abbreviations: 95% CI, 95% confidence interval; BCLC, Barcelona clinic liver cancer; CTLA4, cytotoxic T‐lymphocyte‐associated protein 4; ECOPD, exacerbation of chronic obstructive pulmonary disease; HCC, hepatocellular carcinoma; ICI, immune checkpoint inhibitors; irAE, immune‐related adverse events; NSCLC, non‐small cell lung cancer; ORR, overall response rate; OS, overall survival; PD‐(L) 1, program cell death (ligand) 1; PFS, progression‐free survival; RhA, rheumatoid arthritis.

**FIGURE 2:**
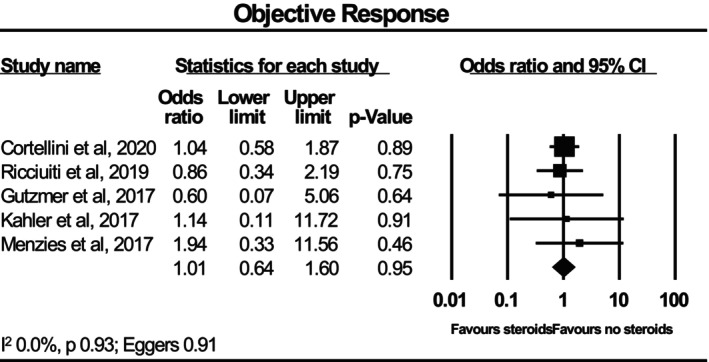
Forest plot for objective response.

Figure 2 shows the meta‐analysis of five studies with a total sample size of 1453 patients for the risk of corticosteroids for non‐cancer‐related indications on objective response rates of immunotherapy. Corticosteroids were not statistically associated with objective response rate, with a pooled odds ratio of 1.01 (95% CI 0.64–1.60). There was no significant heterogeneity, with *I* ^2^ = 0.0%. There was no evidence of publication bias, with Egger's test *p* = 0.91.

**FIGURE 3:**
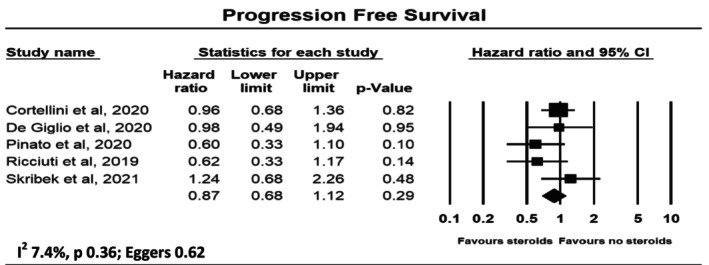
Forest plot for progression free survival.

Figure 3 shows the meta‐analysis of five studies with a total sample size of 2409 patients for the risk of corticosteroids for non‐cancer‐related indications on PFS with immunotherapy. There was no statistically significant association with corticosteroid use, with a pooled hazard ratio of 0.87 (95% CI 0.86–1.12). There was no significant heterogeneity, with *I* ^2^ = 7.4%. There was no evidence of publication bias, with Egger's test *p* = 0.62.

**FIGURE 4:**
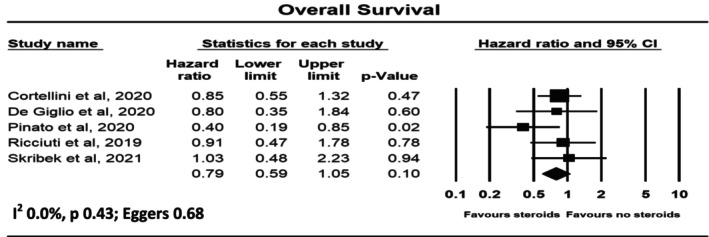
Forest plot for overall survival.

Figure 4 shows the meta‐analysis of five studies with a total sample size of 2409 patients for risk of corticosteroids for non‐cancer‐related indications on OS with immunotherapy. Corticosteroids were not statistically associated with OS, with a pooled hazard ratio of 0.79 (95% CI 0.59–1.05). There was no significant heterogeneity, with *I* ^2^ = 0.0%. There was no evidence of publication bias, with Egger's test *p* = 0.68.

## 4 DISCUSSION

Our systematic review found that there is no difference in response rates or survival outcome for administration of corticosteroids to patients being treated for their malignancy with ICI. It is not possible to conclude from this analysis that corticosteroids do not have any detrimental effect on ICI due to the lack of information regarding doses and length of treatment with corticosteroids. Moreover, high doses of corticosteroids given for prolonged periods are associated with multiple potential complications and should not be considered safe based on this analysis.

These results appear to be congruent with previously published work suggesting that corticosteroids do not affect response to ICI and survival outcome when administered for the management of irAE.^25^, ^26^, ^27^, ^28^, ^29^, ^30^, ^31^, ^32^ The association with irAE however, is reported to potentially be affected by ITB,^43^, ^44^ and is likely to also be confounded by the immunogenic nature of some tumours. ITB can occur in analyses of survival in patients being treated with ICI, as a longer duration of ICI treatment is associated with higher risk of developing irAEs,^52^, ^53^ and therefore by definition will be associated with longer survival, whereas patients with shorter survival and therefore shorter duration of treatment will likely have a lower risk of developing irAE. Multiple studies including Weber et al. and Shimozaki et al. reported a statistically significant longer time on therapy with ICI in patients who experienced an irAE when compared to those who did not experience an irAE.^52^, ^53^ Tang et al. reported on a pooled analysis of 23 clinical trials and 8436 patients. The reported pooled median time for development of grade 3–4 irAE was 4.6–12.2 weeks for CTLA‐4 inhibitors, and 14.1–123.4 weeks for PD‐1/PD‐L1 inhibitors.^54^

In contrast to assessment on the effect of corticosteroids on response to ICI when used for irAE, assessment on the use of corticosteroid for non‐cancer‐related indications should not be affected by ITB, as the use and duration of corticosteroids for non‐cancer‐related indications should not be affected by the use and duration of immunotherapy for cancer. In the papers selected for this analysis, administration of corticosteroids was predominantly for unrelated autoimmune diseases which was reported to be at baseline or prior to initiation of ICI therapy, or for treatment of exacerbation of chronic airway disease which logically should not have any biological relation to ICI therapy.

This analysis does have several limitations however. Firstly, all the studies in our analysis were retrospective, and therefore would have a high risk of being affected by various selection biases. The studies reported on relatively small number of patients treated with corticosteroids for non‐cancer indications, and did not comprehensively report the doses and length of treatment with corticosteroids. Timing of corticosteroid therapy (baseline vs. concurrent) was only mentioned in two studies. These limitations weakens the ability to conclude that any dose of corticosteroids for any length of time is unlikely to harm the response to ICI, as it is possible that the studies only looked at low doses for a short course, and thus might undervalue a possible negative effect of corticosteroids on response to ICI.

Lastly, two studies in our meta‐analysis^38^, ^41^ included patients who received corticosteroids for irAE and could have been affected by ITB, one study did not explicitly detail which indications were considered by the authors as non‐cancer indications^42^ and one study detailed the non‐cancer indications as ‘other inflammation process non‐related to cancer’.^37^ The ambiguity of these definitions could potentially weaken the recommendation as these trials may have potentially included patients with corticosteroid treatment of irAE, thus further enhancing ITB.

Interestingly, Bai et al. reported a retrospective analysis of 509 patients with melanoma who developed irAE. In that study a comparison of survival outcomes was made between patients who required high doses of corticosteroids, defined as more than 60 mg per day of oral prednisolone equivalent, and patients who developed irAEs not requiring high doses of corticosteroids. To minimise ITB the analysis used landmarks of 8 and 26 weeks from initiation of ICI, and patients who had disease progression or died prior to the landmark were excluded from the analysis. This study did report inferior survival parameters for irAE requiring high doses of corticosteroids and is certainly worth consideration.^55^ There is however a potential confounding effect of these results by the possibilities that patients who require higher doses of corticosteroids are likely to have a longer delay to treatment re‐challenge, might be excluded from further ICI altogether or might have developed other complications from immunosuppression that could have a negative effect on survival.

While it is not possible to conclude from the results of this meta‐analysis that high doses for prolonged periods of time will not affect the prognosis of cancer patients treated with ICI, and a specific safety threshold for corticosteroid dosing cannot be established from this analysis, our results do suggest that doses and durations of corticosteroids used commonly for non‐cancer‐related indications do not have a significant effect on response or survival in patients treated with ICI for malignancy. Avoidance of sensible prescribing of corticosteroids for COPD exacerbations, autoimmune disease flares or irAE in the hope of preserving ICI efficacy is therefore not likely to be beneficial.

## 5 CONCLUSION

The results of this meta‐analysis suggest that there is no difference in outcome when corticosteroids are administered concurrently with ICI. This previously reported association of poor response to ICI when co‐administered with corticosteroids is likely to be due to the confounder effect of the indication for which corticosteroids are being prescribed. Corticosteroids are often prescribed to patients with a more aggressive disease, which is in itself a poor prognostic marker with regard to response and survival,^19^ and is therefore the likely explanation for the observed association. The results of this meta‐analysis suggests that there should not be any expected benefit from rapid reduction of doses or avoidance of administration of corticosteroids to patients treated with ICI when indicated, as it is not likely to affect the outcome when prescribed for non‐cancer indications. Corticosteroid administration is similarly unlikely to affect response when prescribed for cancer‐related indications as the main limitation to the effect of ICI is likely to be the disease phenotype, rather than administration of corticosteroids. This analysis has significant limitations with regard to the observational design and size of the studies that were analysed, as well as the lack of details regarding doses, timing and length of treatment with corticosteroids.

## AUTHOR CONTRIBUTIONS

**Yoni Byron:** Conceptualization (equal); data curation (lead); methodology (equal); project administration (equal); writing – original draft (lead); writing – review and editing (equal). **Sonya Yegorova‐Lee:** Data curation (supporting); project administration (equal); writing – original draft (supporting). **Martin Tio:** Conceptualization (equal); formal analysis (lead); methodology (lead); project administration (supporting); supervision (lead); writing – original draft (supporting); writing – review and editing (supporting).

## FUNDING INFORMATION

No funding were received by any of the authors for the purpose of writing this manuscript.

## CONFLICT OF INTEREST STATEMENT

The authors have no conflict of interest to declare.

## DISCLAIMER

The views expressed in the submitted article are those of the authors and not an official position of the institutions employing the authors.

## Data Availability

All materials are available for review through the corresponding author.
